# Melatonin stimulates transcription of the rat *phosphoenolpyruvate carboxykinase* gene in hepatic cells

**DOI:** 10.1002/2211-5463.13007

**Published:** 2020-11-02

**Authors:** Kosuke Asano, Akiko Tsukada, Yuki Yanagisawa, Mariko Higuchi, Katsuhiro Takagi, Moe Ono, Takashi Tanaka, Koji Tomita, Kazuya Yamada

**Affiliations:** ^1^ Department of Health and Nutritional Science Faculty of Human Health Science Matsumoto University Matsumoto Japan; ^2^ Matsumoto University Graduate School of Health Science Matsumoto Japan; ^3^ Laboratory of Molecular Biology Faculty of Pharmacy Osaka Ohtani University Tondabayashi Japan

**Keywords:** dexamethasone, gluconeogenesis, insulin, melatonin, mitogen‐activated protein kinase, phosphoenolpyruvate carboxykinase

## Abstract

Melatonin plays physiological roles in various critical processes, including circadian rhythms, oxidative stress defenses, anti‐inflammation responses, and immunity; however, the current understanding of the role of melatonin in hepatic glucose metabolism is limited. In this study, we examined whether melatonin affects gene expression of the key gluconeogenic enzyme, phosphoenolpyruvate carboxykinase (PEPCK). We found that melatonin treatment increased PEPCK mRNA levels in rat highly differentiated hepatoma (H4IIE) cells and primary cultured hepatocytes. In addition, we found that melatonin induction was synergistically enhanced by dexamethasone, whereas it was dominantly inhibited by insulin. We also report that the effect of melatonin was blocked by inhibitors of mitogen‐activated protein kinase/extracellular signal‐regulated protein kinase (MAPK/ERK), RNA polymerase II, and protein synthesis. Furthermore, the phosphorylated (active) forms of ERK1 and ERK2 (ERK1/2) increased 15 min after melatonin treatment. We performed luciferase reporter assays to show that melatonin specifically stimulated promoter activity of the *PEPCK* gene. Additional reporter analysis using 5′‐deleted constructs revealed that the regulatory regions responsive to melatonin mapped to two nucleotide regions, one between −467 and −398 nucleotides and the other between −128 and +69 nucleotides, of the rat *PEPCK* gene. Thus, we conclude that melatonin induces *PEPCK* gene expression via the ERK1/2 pathway at the transcriptional level, and that induction requires *de novo* protein synthesis.

## Graphical abstract

Our study revealed a new role of melatonin in hepatic glucose metabolism. We examined whether melatonin effects expression of the *phosphoenolpyruvate carboxykinase* (*PEPCK*) gene. Melatonin induced *PEPCK* gene expression via the ERK1/2 pathway at the transcription level, and this induction requires *de novo* protein synthesis.

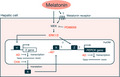

**Abbreviations**
CRE: cAMP response element
CRU: cAMP response unit
Dex: dexamethasone
DMEM: Dulbecco’s modified Eagle’s medium
Erk: extracellular signal‐regulated protein kinase
GRU: glucocorticoid response unit
MAPK: mitogen‐activated protein kinase
PEPCK: phosphoenolpyruvate carboxykinase

The liver plays a central role in blood glucose regulation [1]. During fasting, gluconeogenesis and glycogenolysis are stimulated to produce glucose in the liver, resulting in increased blood glucose [2]. Phosphoenolpyruvate carboxykinase (PEPCK) is a key gluconeogenic enzyme. In rat liver, expression of *PEPCK* gene is increased by glucagon (via cAMP), glucocorticoids (e.g., dexamethasone; Dex), thyroid hormone, and retinoic acid, whereas expression is repressed by insulin [3, 4, 5]; these effects on *PEPCK* gene expression are regulated at the transcriptional level. As the half‐lives of both PEPCK and mRNA are extremely short [6], enzymatic activity of PEPCK reflects transcriptional activity [7].

Melatonin is produced not only in the pineal gland, but also in many other organs in animals [8]. Although melatonin was initially believed to be a hormone found only in animals, it is also produced in most plants and bacteria [9]. Recently, it was determined that melatonin acts as a free radical scavenger toward reducing oxidative stress, and it regulates various biological processes, including circadian rhythm, anti‐inflammation, and immunity [10].

Many people intake melatonin from either food or supplements [11]. Ingested melatonin is absorbed from the intestine and accumulates in the liver via the portal vein. It was reported that oral melatonin administration reduces liver steatosis, hepatic oxidative stress, and mitochondrial dysfunction, suggesting that melatonin regulates the development of obesity and improves lipid metabolism [12, 13, 14]. Stimulation of melatonin to rat hypothalamus represses expression of *PEPCK* and *glucose‐6‐phosphatase* gene in the liver, reduces hepatic gluconeogenesis, and lowers blood glucose [15]. Further, increased melatonin signaling represses insulin secretion, and it promotes glucagon secretion both *in vivo* and *in vitro* [16, 17]. It was also reported that melatonin deficiency induces nocturnal hepatic insulin resistance and increased gluconeogenesis [18]. Therefore, the role of melatonin in blood glucose regulation is unclear, and it remains to be determined whether melatonin directly regulates gluconeogenesis in hepatic glucose metabolism.

In this study, we investigate regulation of *PEPCK* gene expression by melatonin in both rat H4IIE highly differentiated hepatoma cells and primary cultured hepatocytes and identify the underlying molecular mechanism.

## Materials and methods

### Materials

Dulbecco's modified Eagle's medium (DMEM), melatonin, DMSO, and PBS were purchased from Wako Pure Chemical Industries, Ltd (Osaka, Japan). FBS was purchased from BioWest Therapeutics Inc (Nuaillé, France). Streptomycin and penicillin G were purchased from Meiji Seika Pharma Co., Ltd (Tokyo, Japan). Dex and insulin were purchased from Sigma‐Aldrich (Saint Louis, MO, USA). Wortmannin, rapamycin, PD98059, actinomycin D, and cycloheximide (CHX) were purchased from Merck Chemicals Ltd (Darmstadt, Germany). Sepasol‐RNA I Super G and Bullet PAGE One Precast Gel 8% were purchased from Nacalai Tesque Co., Ltd (Kyoto, Japan). High‐Capacity RNA‐to‐cDNA Kit was obtained from Applied Biosystems (Foster City, CA, USA). FastStart Universal SYBR Green Master (ROX) and Genopure Plasmid Maxi Kit were purchased from Roche Diagnostics (Indianapolis, IN, USA). The pGL4.11, the pGL4.13, the phRL‐CMV plasmids, and Dual‐Luciferase Reporter Assay System were obtained from Promega (Madison, WI, USA). The Bio‐Rad Protein Assay was purchased from Bio‐Rad Laboratories (Hercules, CA, USA). Polyvinylidene difluoride (PVDF) membrane and Immobilon Western Chemiluminescent HRP Substrate were purchased from Millipore (Bedford, MA, USA). The phospho‐p44/42 mitogen‐activated protein kinase (MAPK) [extracellular signal‐regulated protein kinase (ERK)1/2] (Thr202/Tyr204) (D13.14.4E XP) (#4370S) and p44/42 MAPK (ERK1/2) (137F5) (#4695S) antibodies were purchased from Cell Signaling Technology (Danvers, MA, USA). The β‐actin (C4) antibody (SC‐47778) was obtained from Santa Cruz Biotechnology (Santa Cruz, CA, USA). Horseradish peroxidase‐conjugated goat anti‐rabbit IgG antibody (#G‐21234) was purchased from Invitrogen (Groningen, the Netherlands). The mouse TrueBlot® ULTRA: Anti‐Mouse Ig HRP (#18‐8817‐31) was obtained from COSMO BIO Co., Ltd (Tokyo, Japan).

### Cells and cell culture

Rat H4IIE hepatoma cells were a generous gift from D. K. Granner (Vanderbilt University, USA). Cells were maintained in DMEM supplemented with 10% FBS and 100 μg·mL^−1^ streptomycin and 100 units per mL penicillin G at 37 °C in a 5% CO_2_ incubator. One million H4IIE cells were seeded in a 6‐cm dish. After 24 h, the medium was replaced with serum‐free DMEM and then cultured for another 24 h. To examine the levels of the *PEPCK* gene expression by melatonin, cells were treated with the indicated concentrations of melatonin for various times.

To analyze the levels of the *PEPCK* gene expression by Dex or insulin and melatonin, cells were treated with 0.5 μm Dex or 10 nm insulin and 1 mm melatonin for various times.

To elucidate signal transduction pathway(s), cells were pretreated with various inhibitors for 30 min and then treated with 1 mm melatonin for another 2 h. 0.2 μm wortmannin, 0.1 μm rapamycin, 25 μm PD98059, 0.8 μm actinomycin D, and 10 μm CHX were used as inhibitors.

### Animals and treatment

Male Sprague Dawley rats (6‐weeks‐olds, 160–180 g body weight) were used. Rats were housed in a photoperiod of 12 h of light and 12 h of darkness (from 20:00 h through 8:00 h). Primary hepatocytes were prepared from the liver by a collagenase perfusion method [19]. This animal experiment was approved by the Matsumoto University Animal Experiment Committee. Treatment of the animals followed Matsumoto University guidelines.

### Real‐time polymerase chain reactions (PCRs)

Total RNA was prepared from various cells using the Sepasol‐RNA I Super G. Procedures were performed according to the manufacture's recommended protocol. Total RNA (0.5 μg) was reverse‐transcribed with High‐Capacity RNA‐to‐cDNA Kit. Reverse transcription real‐time PCRs were previously described [20, 21, 22, 23]. The expression level of PEPCK mRNA was normalized by that of 36B4 mRNA. The nucleotide sequences of primers were as follows: PEPCK forward 5′‐TCCTGATCCTGGGCATAACTAA‐3′, PEPCK reverse 5′‐GTCATCACCCACACATTCAACTTT‐3′, and 36 B4 forward 5′‐GGCGACCTGGAAGTCCAACT‐3′, 36B4 reverse 5′‐GGATCTGCTGCATCTGCTTG‐3′, respectively.

### Western blot analysis

H4IIE cells were treated with or without 1 mm melatonin for 15 min and then collected in PBS. Procedures for preparation of whole‐cell lysates and western blot analysis were previously described [22, 24]. Briefly, whole‐cell lysates (20 μg) were resolved with 8% SDS/PAGE and transferred onto a PVDF membrane for western blot analysis. The phospho‐p44/42 MAPK (ERK1/2) antibody (1 : 2000 dilution), the p44/42 MAPK (ERK1/2) antibody (1 : 1000 dilution), and the β‐actin (C4) antibody were used as the primary antibodies. Horseradish peroxidase‐conjugated goat anti‐rabbit IgG antibody (1 : 20 000 dilution) or horseradish peroxidase‐conjugated goat anti‐mouse IgG antibody (1 : 2000 dilution) was employed as the secondary antibodies. Visualization and analysis of the proteins were also previously described [22, 24].

### Construction of plasmids

The prPEPCK/Luc plasmid which contains a region from −467 to +69 of the rat *PEPCK* gene was previously described [25]. An ~ 530 bp of *Kpn*I/*Bgl*II fragment of the prPEPCK/Luc was subcloned into the *Kpn*I/*Bgl*II sites of the pGL4.11 plasmid to give the pPEPCK/Luc‐467 plasmid. To produce the 5′‐deletion constructs, the pPEPCK/Luc‐467 plasmid was used as the template and PCR was performed. The nucleotide sequences of PCR primers were as follows: −397, 5′‐CCGGACGCGTCCACTGGCACACAAAATGTG‐3′; −224, 5′‐CCGGACGCGTGACACGGGGGCCTGAGGC‐3′; −128, 5′‐CCGGACGCGTGCTGACCATGGCTATGATCC‐3′; and +69, 5′‐CCGGAGATCTCAGAGCGTCTCGC‐3′, respectively.

### Transient DNA transfections and luciferase reporter gene assays

All plasmids were prepared using the Genopure Plasmid Maxi Kit. A calcium phosphate method was employed for transfection into H4IIE cells [26]. To examine an effect of melatonin on promoter activity of the *PEPCK* gene, H4IIE cells were cotransfected with 8 μg of the reporter plasmid and 0.1 μg of the phRL‐CMV indicator plasmid. After 4 h, the transfected cells were incubated with serum‐free DMEM. After 16 h, cells were treated with 1 mm melatonin for various times. In deletion assay, cells were treated with 1 mm melatonin for 6 h.

Firefly and sea pansy luciferase assays were carried out using the Dual‐Luciferase Reporter Assay System. Procedures were performed according to the manufacture's recommended protocol. Luciferase activities were determined by a Berthold Lumat model LB 9507 (Wildbad, Germany). Firefly luciferase activities (relative light units) were normalized by sea pansy luciferase activities.

### Statistical analysis

All experiments were carried out at least three times. Data were represented as the mean and SE and analyzed by one‐way ANOVA followed by Fisher's protected LSD multiple comparison test. In addition, data shown in Fig. 2A were analyzed by two‐way ANOVA in each time.

## Results

### Melatonin induces PEPCK mRNA expression in both H4IIE cells and primary cultured hepatocytes

**Fig. 1:**
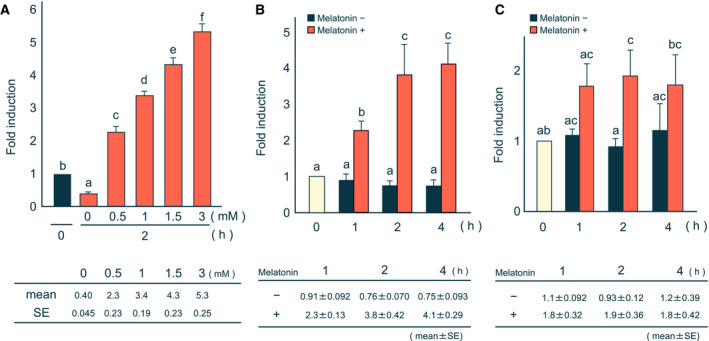
Melatonin induces the PEPCK mRNA levels. Total RNA was prepared from H4IIE cells (A), (B), or rat primary cultured hepatocytes (C) treated with or without melatonin. The levels of PEPCK and 36B4 mRNAs were determined by reverse transcription and quantitative real‐time PCR. Each column and bar represents the mean and SE of the ratio of the levels of PEPCK and 36B4 mRNAs of at least three independent experiments. The expression level ratio shown as ‘0 h’ was normalized to 1. Differences between individual groups were estimated using Fisher’s LSD test. ^a,b,c,d,e,f^ Within each graph, means without a common letter differ, *P* < 0.05. (A) H4IIE cells were treated with the indicated concentrations of melatonin for 2 h. (B) Time course for alterations in the PEPCK mRNA levels by melatonin. H4IIE cells were treated with (+) or without (−) 1 mm melatonin for the indicated times. (C) Rat primary cultured hepatocytes were treated with (+) or without (−) 1 mm melatonin for the indicated times.

First, we examined whether melatonin regulates *PEPCK* gene expression in rat H4IIE hepatoma cells and primary cultured hepatocytes. H4IIE cells are a model cell line that reproduces hormonal regulation of gluconeogenic gene expression in liver. Following 2‐h treatments with different concentrations of melatonin, we found dose‐dependent increases in PEPCK mRNA levels in H4IIE (Fig. 1A). We then examined the time course of PEPCK mRNA induction and found that PEPCK mRNA expression increased 2 h following treatment and that induction was maintained for 4 h (Fig. 1B). Similarly, we found that PEPCK mRNA levels increased 2 h following melatonin treatment in primary cultured hepatocytes (Fig. 1C). These findings indicate that melatonin induces *PEPCK* gene expression in hepatic cells.

### Effects of Dex and insulin on PEPCK mRNA induction by melatonin

**Fig. 2:**
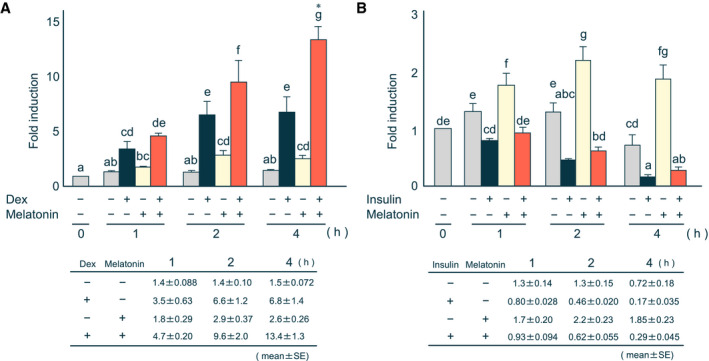
Effects of Dex or insulin on the melatonin induction of PEPCK mRNA levels. Total RNA was prepared from H4IIE cells treated with (A) 0.5 μm Dex or (B) 10 nm insulin in the absence or presence of 1 mm melatonin for the indicated times. Details for determination of the levels of PEPCK mRNA are described in the legend of Fig. 1. The expression level ratio shown as ‘0 h’ was normalized to 1. Each column and bar represents the mean and SE of at least three independent experiments. Differences between individual groups were estimated using Fisher's LSD test. ^a,b,c,d,e,f,g^ Within each graph, means without a common letter differ, *P* < 0.05. * shows an interaction effect (two‐way ANOVA, *P* < 0.05) between Dex and melatonin in each time.

Transcription of *PEPCK* gene is stimulated or repressed by Dex or insulin, respectively [27]. In H4IIE cells, the optimal concentration of Dex and insulin is 0.5 μm and 10 nm, respectively [6, 28]. We examined the effects of these hormones on melatonin induction. PEPCK mRNA was induced in H4IIE cells treated with Dex (Fig. 2A), and the effect of Dex exposure was enhanced in the presence of melatonin (Fig. 2A). Four hours after treatment, we found that PEPCK mRNA levels increased 6.8‐fold with Dex, 2.6‐fold with melatonin, and 13.4‐fold with both hormones, indicating that Dex and melatonin synergistically act on *PEPCK* gene expression (Fig. 2A). In contrast, reduced PEPCK mRNA levels were found in H4IIE cells treated with insulin in the absence or presence of melatonin (Fig. 2B). Based on these findings, Dex and melatonin synergistically induce PEPCK mRNA expression, and that insulin dominantly represses *PEPCK* gene expression even in the presence of melatonin.

### Melatonin induces PEPCK mRNA expression via the MAPK signaling pathway

**Fig. 3:**
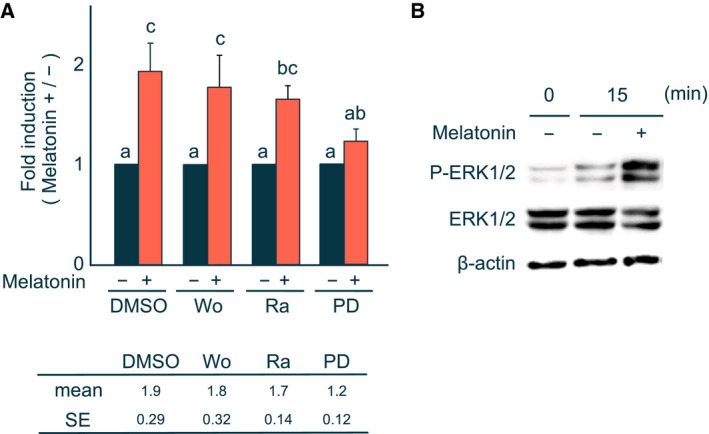
Analysis of signaling pathway mediating the induction of the PEPCK mRNA levels by melatonin. (A) H4IIE cells were pretreated with DMSO, 0.2 μm wortmannin (Wo), 0.1 μm rapamycin (Ra), or 25 μm PD98059 (PD) for 30 min before treatment with (+) or without (−) 1 mm melatonin for 2 h. Details for determination of the levels of PEPCK mRNA are described in the legend of Fig. 1. The value of the ratio in the absence of melatonin in each reagent was set to 1. Each column and bar represents the mean and SE of five independent experiments. Differences between individual groups were estimated using Fisher's LSD test. ^a,b,c^ Within each graph, means without a common letter differ, *P* < 0.05. (B) Whole‐cell lysates were prepared from H4IIE cells treated with (+) or without (−) 1 mm melatonin for 15 min. The anti‐phospho‐p44/42 MAPK (ERK1/2) antibody (top), anti‐p44/42 MAPK (ERK1/2) antibody (middle), or anti‐β‐actin antibody (bottom) was used as the primary antibody, respectively. Three independent experiments were performed, and representative result was shown.

Next, we identified which signaling pathway is involved in melatonin‐mediated PEPCK mRNA induction. H4IIE cells were treated with inhibitors of protein kinases involved in different signal transduction pathways. Administration of wortmannin, a phosphoinositide 3‐kinase inhibitor, or the p70S6 kinase inhibitor rapamycin had no effect on PEPCK mRNA levels in H4IIE cells; however, its expression was blocked by the administration of the MAPK/ERK kinase (also known as MEK) inhibitor PD98059 (Fig. 3A). Thus, the effect of melatonin on PEPCK mRNA induction is mediated by the MAPK pathway.

To explore activation of ERK1 and ERK2 (ERK1/2) by melatonin, whole‐cell lysates were prepared from H4IIE cells treated with or without melatonin and subjected to western blot analysis. We found that phosphorylated (active) forms of ERK1/2 were detected 15 min after melatonin treatment (Fig. 3B). In contrast, levels of the nonphosphorylated forms of ERK1/2 were practically unchanged. These results indicate that melatonin rapidly activates ERK1/2 in H4IIE cells.

### Induction of PEPCK mRNA by melatonin is regulated at the transcriptional level and dependent on *de novo* protein synthesis

**Fig. 4:**
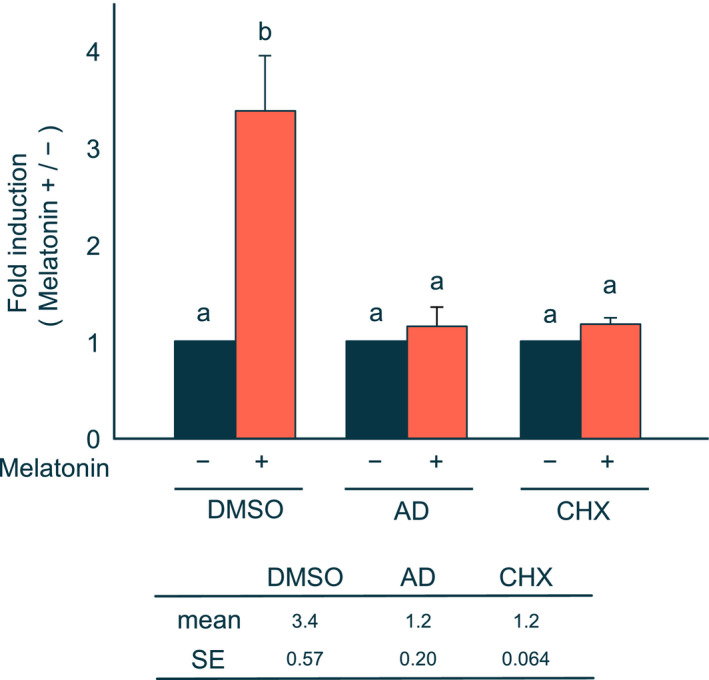
Effects of actinomycin D or CHX on melatonin‐induced PEPCK mRNA levels. H4IIE cells were pretreated with DMSO, 0.8 μm actinomycin D (AD), or 10 μm CHX for 30 min before treatment with (+) or without (−) 1 mm melatonin for 2 h. Details for determination of the levels of PEPCK mRNA are described in the legend of Fig. 1. The value of the ratio in the absence of melatonin in each reagent was set to 1. Each column and bar represents the mean and SE of at four independent experiments. Differences between individual groups were estimated using Fisher's LSD test. ^a,b^ Within each graph, means without a common letter differ, *P* < 0.05.

To determine whether induction of PEPCK mRNA by melatonin requires *de novo* RNA and protein synthesis, H4IIE cells were pretreated with actinomycin D and CHX, an inhibitor of RNA polymerase II and protein synthesis, respectively. We found that induction was completely inhibited by both compounds (Fig. 4). Thus, our findings suggest that the induction of PEPCK mRNA by melatonin occurs at the transcriptional level of *PEPCK* gene, and that induction requires *de novo* protein synthesis.

### Melatonin increases promoter activity of rat *PEPCK* gene

**Fig. 5:**
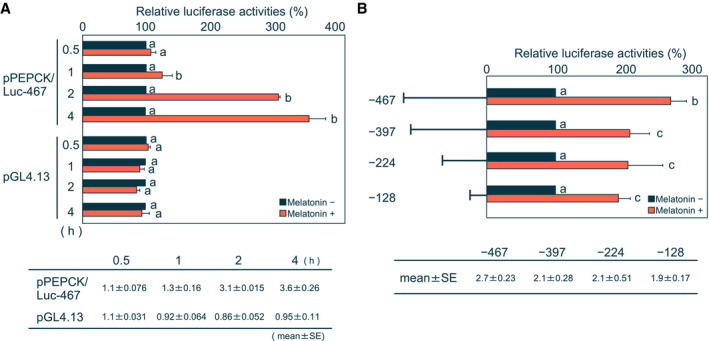
Melatonin stimulates the transcriptional activity of the rat *PEPCK* gene. The transfected cells were cultured for 18 h and then treated with (+) or without (−) 1 mm melatonin for 6 h. A value of 100 was assigned to promoter activity from (A) each reporter plasmid at each time or (B) each reporter plasmid, in the absence of melatonin. Each column and bar represents the mean and SE of the value of the PEPCK promoter activity of at least three independent experiments. Differences between individual groups were estimated using Fisher's LSD test. ^a,b,c^ Within each graph, means without a common letter differ, *P* < 0.05. (A) The pPEPCK/Luc‐467 or the pGL4.13 plasmid was used as the reporter plasmid. (B) The pPEPCK/Luc‐467 (top) and the 5′‐deletion constructs including the pPEPCK/Luc‐397 (the second of the top), the pPEPCK/Luc‐224 (the third of the top), and the pPEPCK/Luc‐128 (bottom) were used as the reporter plasmid, respectively.

We also evaluated the effect of melatonin on promoter activity of rat *PEPCK* gene using a luciferase reporter gene assay. The region between −467 and +69 nucleotides of rat *PEPCK* gene contains all elements required for hepatic expression and hormonal regulation [4, 29, 30]. A pPEPCK/Luc‐467 reporter plasmid containing this region was transfected into H4IIE cells with an indicator plasmid. Cells were then treated with melatonin for various durations. We found that promoter activity of this plasmid was increased by melatonin treatment (Fig. 5A). In contrast, we found no change in activity with a pGL4.13 plasmid containing the SV40 enhancer/promoter region following melatonin administration (Fig. 5A). These findings indicate that melatonin specifically enhances transcriptional activity of *PEPCK* gene.

Next, to elucidate the melatonin‐responsive region of *PEPCK* gene, we prepared three 5′‐deletion constructs: pPEPCK/Luc‐397, pPEPCK/Luc‐224, and pPEPCK/Luc‐128, containing nucleotide sequences between −397 and +69, −224 and +69, and −128 and +69 nucleotides of rat *PEPCK* gene, respectively. Following transfection into H4IIE cells, promoter activity of all three constructs was increased by melatonin (Fig. 5B). However, we found that the ratios were significantly lower compared with that of pPEPCK/Luc‐467 plasmid (Fig. 5B). Based on these findings, we determined that melatonin‐responsive regions are located in nucleotide regions −467 to −398 and −128 to +69 of the *PEPCK* gene.

## Discussion

In this study, to investigate the role of melatonin in hepatic gluconeogenesis, we explored whether melatonin regulated *PEPCK* gene expression, and found that melatonin increased mRNA levels of PEPCK in both rat H4IIE cells and primary cultured hepatocytes (Fig. 1). Thus, our findings show that melatonin has a role in regulating blood glucose, which is not unexpected as melatonin is secreted at night when humans are in a fasting state. In fact, recent studies have reported that enhanced melatonin signaling caused by mutations in melatonin receptors suppresses insulin secretion in pancreatic beta cells [16], suggesting that melatonin inhibits insulin secretion and prevents nocturnal hypoglycemia. Further, melatonin promotes glucagon secretion *in vivo* and *in vitro* [17]. We also examined whether hormones regulating blood glucose affect the melatonin‐mediated induction, and found that Dex and melatonin synergistically increase PEPCK mRNA levels, suggesting that the mechanism underlying Dex‐mediated induction of *PEPCK* gene expression partially cross‐talks between that of melatonin. Insulin represses stimulation of *PEPCK* gene transcription by other hormones and retinoic acid [6]. Similarly, we found that insulin dominantly represses induction of *PEPCK* gene expression by melatonin (Fig. 2B). In addition, melatonin‐mediated induction of PEPCK mRNA levels was inhibited by PD98059, a MAPK signaling pathway inhibitor (Fig. 3A). There are two kinds of melatonin receptors that belong to the G protein‐coupled receptor family and have Gi activity [31]. It was reported that the Gi receptor type activates MAPK, and that melatonin induces phosphorylation of ERK1/2 in GTI‐7 cells, rapidly inducing expression of c‐fos and c‐jun mRNA within 30 min [32, 33]. Similarly, we found that ERK1/2 are activated by melatonin in H4IIE cells (Fig. 3B); however, our results do not exclude the possibility that other signaling molecules are involved in the induction of PEPCK mRNA levels by melatonin as they were obtained from experiments with inhibitors and not tailored siRNAs.

Our findings demonstrate that induction of *PEPCK* gene expression by melatonin occurs at the transcription level and requires *de novo* protein synthesis (Fig. 4). Further, promoter activity of the *PEPCK* gene was specifically stimulated by melatonin (Fig. 5A). A region between −467 and +69 nucleotides of the *PEPCK* gene contains two positive transcriptional units, a glucocorticoid response unit (GRU) and a cAMP response unit (CRU). The GRU consists of two glucocorticoid receptor‐binding sites (GR1 and GR2), three accessory factor‐binding sites (AF1, AF2, and AF3), and a cAMP response element (CRE) [29, 34, 35], whereas the CRU consists of a CRE and P3 element [36, 37, 38, 39, 40]. Melatonin‐responsive regions of *PEPCK* gene were mapped to nucleotide regions between −467 and −398 and between −128 and +69 nucleotides (Fig. 5B). Nucleotide sequences between −467 and −398 contain AF1 and AF2 of the GRU, and nucleotide sequences between −128 and +69 contain the CRE. It remains to be determined whether these elements are responsive to melatonin. Chicken ovalbumin upstream promoter transcription factor II, hepatocyte nuclear factor 4, and retinoic acid receptor/retinoid X receptor bind to AF1 elements and forkhead box O1 binds to AF2 elements [4], whereas cAMP‐responsive element‐binding protein and CAAT/enhancer‐binding proteins bind to the CRE. Previously, it was found that some of these transcription factors are activated by the MAPK signaling pathway [41, 42, 43, 44, 45]. Therefore, it is possible that melatonin activates one or more of these transcription factors and/or an unknown transcription factor(s), thereby stimulating transcription of *PEPCK* gene.

The effect of melatonin on *PEPCK* gene transcription may be responsible for its functional properties as an antioxidant rather than a hormone. Previous studies reported that increased concentrations of melatonin act as an antioxidant or antiangiogenic factor in H4IIE and HepG2 hepatoma cells [46, 47, 48]. Further, other studies on the therapeutic effect of melatonin on experimental acute porphyria suggested that melatonin enhances PEPCK activity by scavenging reactive oxygen species that peroxidize lipids [49]. Melatonin is absorbed from the intestine and accumulates in the liver via the portal vein. As our findings suggest that melatonin increases blood glucose levels by facilitating gluconeogenesis, people consuming large oral doses of melatonin as a supplement may accumulate excessive amounts of melatonin in their liver, causing increased levels of blood glucose. Thus, excessive melatonin intake may confer an additional risk for individuals with diabetes who need stricter control of blood glucose levels. Therefore, our findings not only provide a basis for understanding the risk of elevated blood glucose levels because of excessive melatonin intake, but also toward determining the recommended intake for at‐risk groups.

We conclude that melatonin induces expression of rat *PEPCK* gene at the transcriptional level via activation of ERK1/2 and that protein synthesis is required for gene induction. In addition, we identified melatonin‐responsive elements in two nucleotide regions between −467 and −398 and between −128 and +69 nucleotides of the *PEPCK* gene. Furthermore, our study is the first demonstration of transcriptional stimulation of rat *PEPCK* gene in both H4IIE cells and primary cultured hepatocytes by melatonin. To further elucidate its molecular mechanism, additional studies are warranted toward the identification and characterization of multiple melatonin‐responsive elements and binding factors.

## Conflict of interest

The authors declare no conflict of interest.

## Author contributions

KY conceived and designed the project. KA, AT, YY, and MH acquired the data. KA, AT, KTA, MO, TT, KTO, and KY analyzed and interpreted the data. KA, AT, and KY wrote the paper.

## Data Availability

Data and details of the analyses are available from the corresponding author upon reasonable request.
